# Exploratory Evaluation of Neopterin and Chitotriosidase as Potential Circulating Biomarkers for Colorectal Cancer

**DOI:** 10.3390/biomedicines11030894

**Published:** 2023-03-14

**Authors:** Andra Ciocan, Răzvan A. Ciocan, Nadim Al Hajjar, Andreea M. Benea, Stanca L. Pandrea, Cristina S. Cătană, Cristina Drugan, Valentin C. Oprea, Dan S. Dîrzu, Sorana D. Bolboacă

**Affiliations:** 1 Department of Surgery—Surgery Clinic III, “Iuliu Hațieganu” University of Medicine and Pharmacy Cluj-Napoca, 400006 Cluj-Napoca, Romania; 2 “Prof. Dr. Octavian Fodor” Regional Institute of Gastroenterology and Hepatology Cluj-Napoca, 400006 Cluj-Napoca, Romania; 3 Department of Surgery—Practical Abilities, “Iuliu Hațieganu” University of Medicine and Pharmacy Cluj-Napoca, 400006 Cluj-Napoca, Romania; 4 County Emergency Clinical Hospital Cluj-Napoca, 400006 Cluj-Napoca, Romania; 5 Department of Microbiology, “Iuliu Haţieganu” University of Medicine and Pharmacy Cluj-Napoca, 400349 Cluj-Napoca, Romania; 6 Department of Medical Biochemistry, “Iuliu Haţieganu” University of Medicine and Pharmacy Cluj-Napoca, 400349 Cluj-Napoca, Romania; 7 Department of Surgery—Surgery Clinic II, “Iuliu Hațieganu” University of Medicine and Pharmacy Cluj-Napoca, 400006 Cluj-Napoca, Romania; 8 “Dr. Constantin Papilian” Military Emergency Hospital Cluj-Napoca, 400132 Cluj-Napoca, Romania; 9 Department of Medical Informatics and Biostatistics, “Iuliu Hațieganu” University of Medicine and Pharmacy Cluj-Napoca, 400349 Cluj-Napoca, Romania

**Keywords:** colorectal cancer, chitotriosidase, neopterin, biomarkers, systemic inflammation

## Abstract

Chronic inflammation is demonstrated to play a direct role in carcinogenesis. Our exploratory study aimed to assess the potential added value of two inflammation biomarkers, chitotriosidase and neopterin, in follow-up evaluation of patients with colorectal cancer (CRC). An observational exploratory study was conducted. Patients with CRC and matched controls (1:1, age, sex, and living environment) were evaluated. The patients with CRC (CRC group) and controls were assessed at baseline (before surgical intervention for patients with CRC). Patients with CRC were also evaluated at 1-year follow-up. Significantly more patients with blood group A (54.5% vs. 25.0%) and smokers (50.0% vs. 22.7%) were in the CRC group. The serum values of chitotriosidase and neopterin were higher in CRC patients than in controls, but only neopterin reached the conventional level of statistical significance (*p*-value = 0.015). The circulating chitotriosidase and neopterin values decreased significantly at 1-year follow-up (*p*-value < 0.0001). Patients with higher N- and M-stage showed statistically significant higher levels of chitotriosidase and neopterin at baseline and 1-year follow-up (*p*-values < 0.03). Circulating chitotriosidase levels also showed statistically significant differences regarding baseline and 1-year follow-up on patients with CRC and different differentiation grades (*p*-values < 0.02). The circulating levels of neopterin significantly decreased at 1-year follow-up, indicating its potential as a prognostic marker. The circulating values of chitotriosidase and neopterin exhibit significant differences in patients with than without recurrences. Our results support further evaluation of chitotriosidase and neopterin as prognostic markers in patients with CRC.

## 1. Introduction

Colorectal cancer (CRC) is the third most common type of cancer worldwide, 3rd most common in men, and 2nd most in women [1]. Colorectal cancer was responsible in 2020 for 12.7% of all new cancer diagnoses in 27 European Countries and 12.4% of all deaths due to cancer [1]. 

Tumor development and tumor progression are associated with chronic inflammation, impaired immunity, and cellular activation [2,3]. Moreover, in a mutual and continuous exchange of information, tumor cells are exposed to microenvironment transformation [4]. The presence of lymphocyte cells is noticed at the microscopic level, with tumor-associated macrophage cells as a significant component of tumor infiltrates [5]. Tumor-associated macrophage cells are involved in tumor progression by stimulating angiogenesis, tumor proliferation, invasion, and metastasis [6]. Colonization of the gut with proinflammatory bacterial strains, interpreted as dysbiosis, promotes chronic inflammation. Furthermore, the interaction between the intestinal microbiome and the immune system acts as aggressive elements on the gut mucosa, thus increasing the risk of dysplasia and consecutive carcinogenesis [7,8,9]. 

Neopterin is a direct product of the immune system activation, stimulated by the T-cell’s release of interferon-γ, which also induces indoleamine 2,3-dioxygenase, an enzyme involved in the catabolism of tryptophan to kynurenine [10]. Neopterin is the oxidized form of 7,8-dihydroneopterin, a catabolite of the purine nucleotide guanosine triphosphate—GPT [11]. The effects of neopterin are not yet fully elucidated. Still, studies indicated a link between neopterin and oxidative stress, with the formation of reactive oxygen species [12] known to play an essential role in the initiation, proliferation, and development of cancer cells by maintaining their survival [13]. The serum values of neopterin showed limited performance as a marker for CRC compared to CEA (CarcinoEmbryonic Antigen), TPA (tissue plasminogen activator), and CA 19/9 (cancer antigen 19-9) [14]. However, patients with CRC showed significantly elevated neopterin levels (median = 20.2 nmol/L, IQR = [14.2 to 27.2], where IQR is the interquartile range) than controls (median = 19.6 nmol/L, IQR = [15.4 to 24.2]; *p*-value < 0.001) [15]. Furthermore, Zuo et al. [16] reported that subjects with elevated serum neopterin levels are at higher risk of developing colorectal cancer (hazard ratio = 1.09, 95% CI = [1.03 to 1.16], *p* = 0.007; value adjusted for age, sex, body mass index, smoking status, and renal function). 

Chitotriosidase, an enzyme produced by polymorphonuclear neutrophils and mature macrophages in their late differentiation state [17], is encoded by the CHIT1 gene on chromosome 1q32.1 [18]. It is a well-conserved enzyme that catalyzes the hydrolysis of chitin and chitin-like substrates [19], aiding the destruction of chitotriose-walled pathogens and, thus, promoting innate immunity [20]. The enzyme is closely related to macrophage and neutrophilic activation. Chitotriosidase serum levels had higher values in patients with critical limb ischemia [21], diabetes mellitus [22], lysosomal storage disorders [23], overweight and obesity in children [24], breast or prostate cancer [20], and pulmonary diseases [25,26]. Patients with CRC had higher levels of chitinase (median = 21.13 ng/μL, IQR = (17.35–26.16)) than healthy controls (median = 17.21 ng/μL, IQR = (15.39–21.27); *p* < 0.0001) [27]. Furthermore, metastasis was associated with higher chitinase levels in colorectal cancer patients. Kawada et al. [28] reported significantly (*p*-value < 0.05) higher plasma levels of CHI3L1 (chitinase 3-like 1), another member of the family of chitinases, in patients with CRC (*n* = 31) than in controls (*n* = 12), the higher values being associated with TNM (T = tumor, N = nodes, and M = metastases) stage III/IV [28]. 

We hypothesize that circulating levels of chitotriosidase and neopterin, as biomarkers of inflammation, might change in patients with colorectal cancer after surgery, followed by oncological treatment or not. The objectives of the current study were to assess: (i) the variability of circulating chitotriosidase and neopterin in patients with CRC as compared to controls; (ii) the changes of circulating values of chitotriosidase and neopterin after standard treatment in patients with CRC; (iii) the association of chitotriosidase and neopterin plasma levels with current CRC markers.

## 2. Materials and Methods

The study was conducted according to the ethical principles of the Declaration of Helsinki. All patients signed informed written consent at inclusion in the study after an appropriate presentation of study objectives and highlighting the volunteer participation and the right to withdraw without consequences upon the medical care.

### 2.1. Study Design

**Figure 1:**
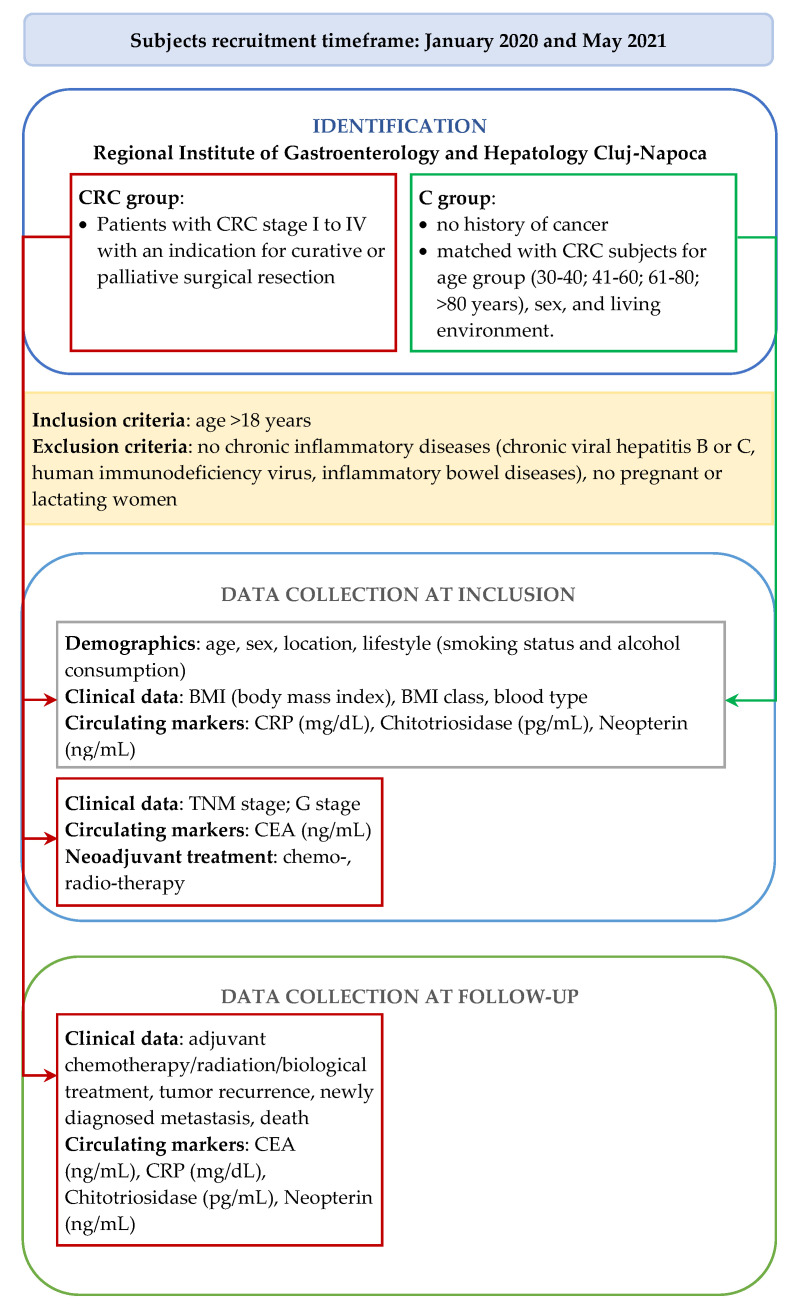
Flowchart of study design. CRP—C-Reactive Protein; CEA—CarcinoEmbryonic Antigen.

An exploratory observational study was conducted at Third Surgical Clinic, “Prof. Dr. Octavian Fodor” Regional Institute of Gastroenterology and Hepatology Cluj-Napoca, Romania. Two groups of subjects were evaluated: the CRC group, which included patients with a diagnosis of CRC based on a colonoscopy with a positive adenocarcinoma diagnosis at biopsy, and the control group (C group), which included matched (1:1) CRC-free subjects (Figure 1). The stage of CRC was established according to contrast-enhanced Computer Tomography (CT) scan, Contrast Enhanced Ultrasound (CEUS), or Magnetic Resonance Imaging (MRI) [29]. 

### 2.2. Demographic and Clinical Characteristics

Age, sex, location, and lifestyle (smoking status and alcohol consumption) data were collected as demographic variables. Data regarding body mass index (BMI) and blood type, known risk factors for CRC [30,31], were used to characterize the subjects included in the study.

### 2.3. Measurement of Circulating Biomarkers

Peripheral venous blood samples were collected from participants at the inclusion, and the circulating markers were dosed (Figure 1). Blood samples were centrifuged within less than 45 min from the collection to obtain 1.5 mL of serum, which was later frozen and stored at −80 °C. The biochemical dosages of the inflammation markers (chitotriosidase and neopterin) took place using the Human Neopterin ELISA kit Fine Test^®^ (EH3413, Wuhan Fine Biotech Co., Wuhan, China) and Human CHIT1 (Chitotriosidase-1) ELISA kit Elabscience^®^ (E-EL-H5620, Elabscience Biotechnology Inc, Houston, TX, USA), according to the manufacturer’s instructions.

To quantify C-Reactive Protein (CRP), particles with a polystyrene core and a hydrophilic shell are employed to connect anti-CRP antibodies covalently. A diluted test sample solution is combined with latex particles coated with monoclonal anti-CRP antibodies from mice. The CRP present in the test sample will combine with latex particles to produce an antigen-antibody complex. Light scattering, determined by a nephelometric technique after six minutes, is proportional to the sample’s analyte concentration. A blank subtraction is executed automatically, and the CRP concentrations are calculated using a calibration curve. Signal data reduction is conducted using a logit-log function for the stored calibration curve. For quantitative CRP determination, these experiments were done using a Behring Nephelometer.

The concentration of CarcinoEmbryonic Antigen (CEA) was measured using a semi-quantitative fluorescence approach. Establishing a CEA Standard Curve CEA antigen samples of varying concentrations were sequentially added and treated with tagged primary antibodies and fluorescence-labeled secondary antibodies. The microfluidic device was spun in a horizontal centrifuge for 150 s at 2500 rpm. Simultaneously, fluorescence pictures were captured using a microscope with an exposure period of 3.5 s. ImageJ software was used to build a standard curve between the concentration of CEA and the matching fluorescence intensity from the fluorescence pictures. Bovine serum albumin (BSA) blocked bead–antibody complexes, labeled primary antibodies, and fluorescence-labeled secondary antibodies were introduced to the microfluidic centrifuge system and incubated at room temperature for 2 h. Then, clinical blood samples obtained from healthy and cancer patients were then put into the centrifuge chip and spun for 2.5 min at 2500 rpm. After acquiring fluorescence pictures using a fluorescence microscope and processing them with the ImageJ program, the standard concentration curve was used to calculate the experimental CEA concentrations.

Measurements of CRP (C-Reactive Protein) and CEA (CarcinoEmbryonic Antigen) were made with COBAS PRO C 503/E801 (Roche Diagnostics International Ltd., Rotkreuz, Switzerland). 

Circulating markers of patients with CRC were measured at the 1-year follow-up visit. Furthermore, the events that occurred between the surgery and the 1-year follow-up were collected: adjuvant chemotherapy/radiation/biological treatment, tumor recurrence, newly diagnosed metastasis, and vital status (dead/alive). 

### 2.4. Statistical Methods

Qualitative raw data were reported as numbers and ratios or percentages, and the differences between groups were tested with the Chi-squared or Fisher’s exact test according to expected frequencies. The distribution of quantitative data was tested with the Shapiro-Wilk test and reported as mean (standard deviation) whenever *p*-value > 0.05, or median IQR (interquartile range, defined as [Q1 to Q3], where Q1 is the value of the first quartile and Q3 is the value of the third quartile). The differences between groups (CRC group vs. C group) on quantitative data were tested with a Student t-test for data that proved to follow the normal distribution; otherwise, the Mann-Whitney test was used. The Kruskal-Wallis test was used to compare more than two sub-groups at once (e.g., T-stage, G grade, etc.) followed by post-hoc analysis whenever statistical significance was observed and more than three subjects per sub-group were encountered. Wilcoxon matched pairs test was applied to compare the baseline with follow-up quantitative data in the CRC group. 

The exploratory statistical analysis was run with the TIBCO Statistica program (v. 13.5, StatSoft Inc, Tusla, OK, USA) at a significance level of 5%.

## 3. Results

Fifty-nine patients with CRC were eligible, and 44 patients were evaluated. Fifteen patients were lost from postoperative follow-up and thus were excluded from the study.

### 3.1. Colorectal Cancer Group vs. Control Group

Forty-four patients with CRC and an equal number of cancer-free subjects aged between 31 and 86 years were evaluated. 

Most evaluated subjects were men (29, 65.9%), and a small number of participants were from rural areas (16 subjects, 36.4%) in each group. The groups were similar regarding age, declared alcohol consumption, and body mass index (BMI) (Table 1). The colorectal cancer group contains significantly more smokers and patients with blood type AII (Table 1).

Twenty-two subjects in the CRC group were smokers, out of which 5 (5/22) were light smokers, 8 (8/22) were moderate smokers, and 9 (9/22) were heavy smokers. Ten subjects in the C group were smokers: 1 (1/10) light smoker, 3 (3/10) moderate smokers, and 6 (6/10) heavy smokers. No significant association was observed between smoking status (light, moderate, or heavy smokers) and the groups (Fisher’s exact test: *p*-value = 0.6495).

**Figure 2:**
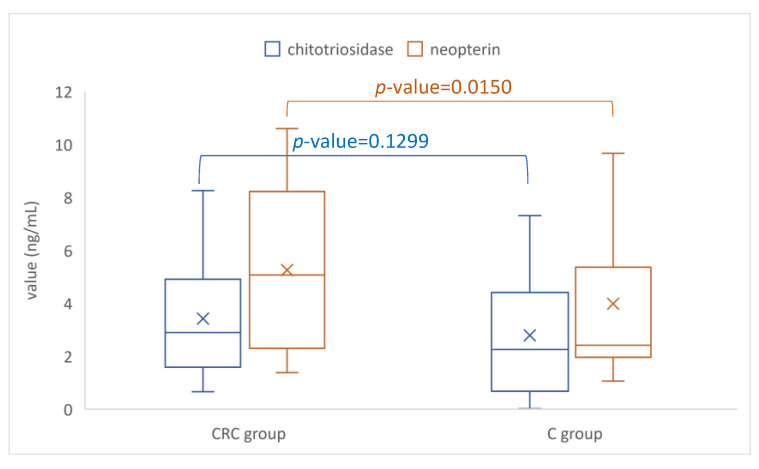
Chitotriosidase and neopterin by groups (CRC = colorectal cancer; C = control). The × in the box indicates the mean value, the line in the box corresponds to the value of the median, the lower and upper bound of the box corresponds to the value of the first (lower) and third (upper) quartile, and the whiskers correspond to the values of minimum and maximum.

The values of CRP and neopterin were significantly higher in the CRC group than in the C group (Table 2 and Figure 2).

The circulating value of chitotriosidase and neopterin showed no significant differences when smokers were compared with non-smokers, neither in the CRC group (Mann-Whitney test: *p*-value = 0.9626 for chitotriosidase and 0.6221 for neopterin) nor in C group (Mann-Whitney test: *p*-value = 0.9219 for chitotriosidase and 0.2686 for neopterin).

### 3.2. Patients with Colorectal Cancer: Pre- and Postoperative Comparison

Most tumors in the colorectal cancer group were moderately differentiated grade G2 and metastasis-free (Table 3).

A total of two patients from the colorectal cancer group (T4 stage) died after discharge during the postoperative follow-up. Therefore, the number of patients in the follow-up comparison was 42. 

The values of CEA significantly reduced at follow-up in patients without recurrent metastasis (Table 4). The serum values of CRP at 1-year follow-up were not statistically significant different from the baseline (Table 4).

Thirty-two out of forty-two patients (76.2%) received adjuvant chemotherapy. Twenty-five patients received neoadjuvant radiotherapy (56.8%), 28 received chemotherapy (63.6%), and six patients (13.9%) received biological treatment. The hospitalization stays ranged from 6 to 24 days (median = 9, IQR = [7 to 11]). Tumor recurrence was observed in 8 patients (19%), and metastasis in evolution was observed in 10 patients (23.8%).

**Figure 3:**
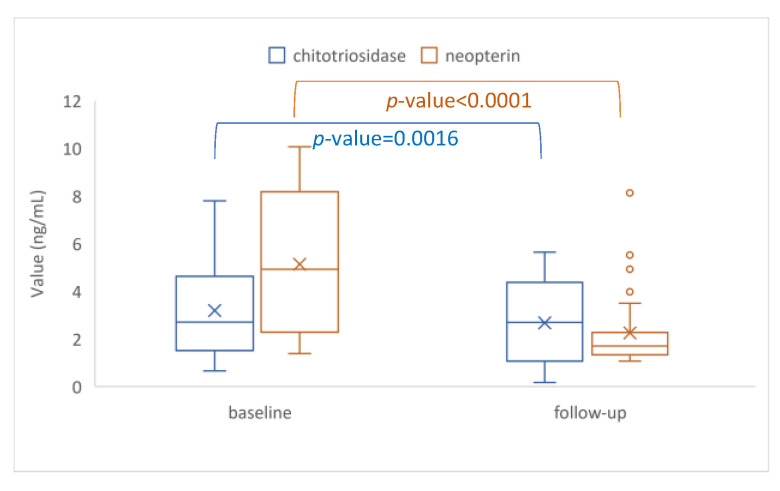
Chitotriosidase and neopterin: postoperative changes on patients with CRC. The × in the box indicates the mean value, the line in the box corresponds to the value of the median, the lower and upper bound of the box corresponds to the value of the first (lower) and third (upper) quartile, and the whisker corresponds to the values of minimum and maximum. The extreme values are shown as “o”.

The values of chitotriosidase and neopterin decreased significantly at follow-up (Figure 3). The circulating values of neopterin remain significantly reduced at 1-year follow-up after excluding extreme values (Wilcoxon matched pairs test: *p*-value < 0.0001). 

Neopterin proved to be sensitive in distinguishing between tumor stages at baseline, N-stage, and M-stage, both baseline and follow-up (Table 5). Similar differences were also observed for chitotriosidase; this marker was also significantly associated with the differentiation grade (Table 5).

**Figure 4:**
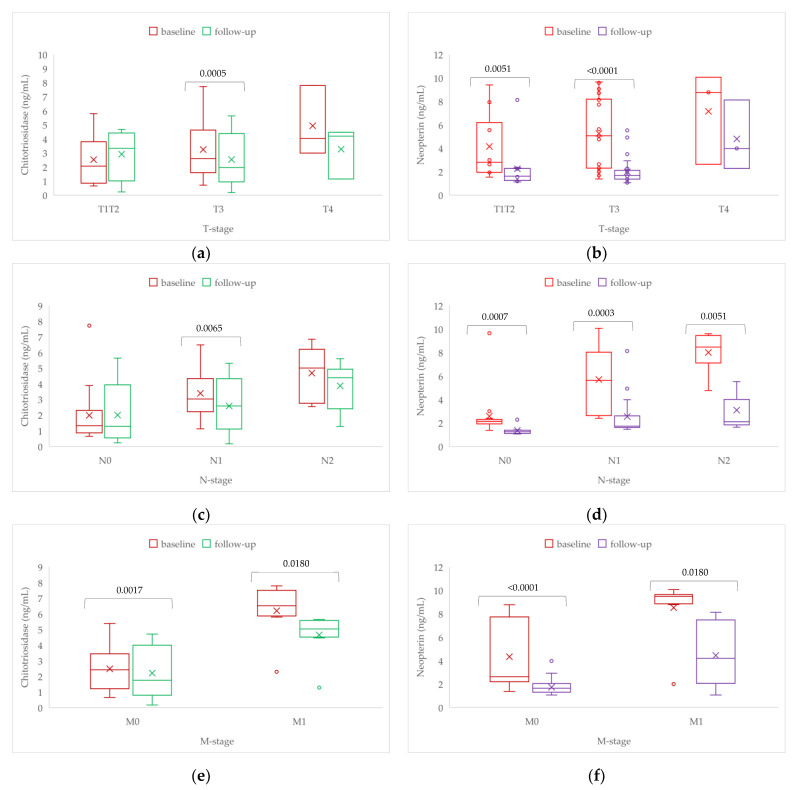
Baseline vs. 1-year follow-up chitotriosidase and neopterin differences according to (**a**) and (**b**) T-stage; (**c**) and (**d**) N-stage; (**e**) and (**f**) M-stage. The × in the box indicates the mean value, the line in the box corresponds to the value of the median, the lower and upper bound of the box corresponds to the value of the first (lower) and third (upper) quartile, and the whisker corresponds to the values of minimum and maximum. The extreme values are shown as “o”. The differences between baseline and follow were tested with Wilcoxon matched pairs test.

**Figure 5:**
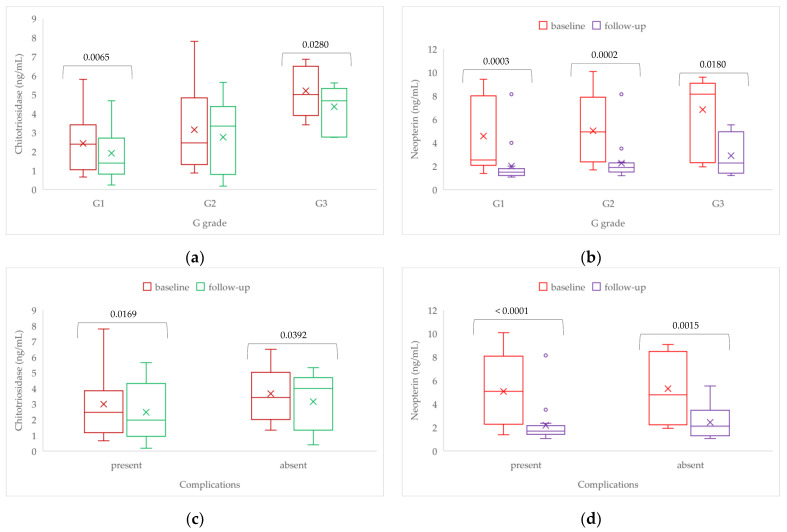
Baseline vs. follow-up chitotriosidase and neopterin differences according to (**a**) and (**b**) differentiation grade (G-grade); (**c**) and (**d**) presence or absence of complications. The × in the box indicates the mean value, the line in the box corresponds to the value of the median, the lower and upper bound of the box corresponds to the value of the first (lower) and third (upper) quartile, and the whisker corresponds to the values of minimum and maximum. The extreme values are shown as “o”. The differences between baseline and follow were tested with Wilcoxon matched pairs test.

The variation of chitotriosidase and neopterin and significant differences within sub-groups are illustrated in Figure 4 and Figure 5.

No statistically significant differences were observed when baseline values were compared with 1-year follow-up values neither for chitotriosidase nor for neopterin in patients with and without neoadjuvant radiotherapy (Mann-Whitney tests: *p*-values > 0.18) or chemotherapy (Mann-Whitney tests: *p*-values > 0.28). Patients with biological neoadjuvant therapy exhibit, at the baseline measurements, significantly higher values of chitotriosidase (with 6.7 ng/mL [6.5 to 7.5], *n* = 6 vs. without 2.5 ng/mL [1.3 to 3.6], *n* = 37, Mann-Whitney test: *p*-value = 0.0002) and neopterin (with 9.6 ng/mL [9.5 to 9.6], *n* = 6 vs. without 2.6 ng/mL [2.2 to 7.8], *n* = 37, Mann-Whitney test: *p*-value = 0.0001) than those without biological neoadjuvant therapy.

Patients with tumor recurrence at 1-year follow-up showed statistically significant elevated follow-up chitotriosidase values (with 4.3 ng/mL [4.1 to 4.7], *n* = 8 vs. without 1.8 ng/mL [0.9 to 4], *n* = 34; Mann-Whitney test: *p*-value = 0.0282) and neopterin (with 3.1 ng/mL [2.3 to 6.2], *n* = 8 vs. without 1.6 ng/mL [1.3 to 2], *n* = 34, Mann-Whitney test: *p*-value = 0.0003) than those without recurrence.

A monotonic association has been identified between circulating values of chitotriosidase and CEA at the 1-year follow-up, with a value of 0.30 for Spearman’s correlation coefficient (ρ) (*p*-value = 0.0496). The association between the two at baseline reached a tendency to statistical significance (ρ = 0.27, *p*-value = 0.0780).

## 4. Discussion

Our study showed increased circulating levels of chitotriosidase and neopterin in patients with colorectal cancer compared to cancer-free subjects. Still, the difference reached statistical significance only for neopterin. Chitotriosidase and neopterin showed high and proportional levels in patients with CRC with advanced stages and with the presence of metastasis. At the 1-year follow-up, a statistically significant decrease in neopterin circulating levels was observed, indicating its potential as a prognostic marker.

### 4.1. Patients with Colorectal Cancer versus Controls

The subjects included in our study were between 31 and 86 years old, with a higher preponderance of men (65.9%) as already reported in the scientific literature [32]. The median age of patients with CRC in our study was 63 years. Studies have shown an increasing trend in incidence towards young ages, between 20 and 40 years, compared to previous reports [33,34], probably due to the screening programs. 

In our sample, half of the patients in the CRC group were smokers, almost twice that in the control group (Table 1). The association between smoking and CRC is already known, with a statistically significant pool relative risk of 1.18 (IQR = 1.11 to 1.25) reported by Botteriet al. [35]. Smoking was associated with a poor prognosis in colorectal cancer (pooled estimated relative risk of cancer mortality equal to 1.25, IQR = 1.14 to 1.37) [35]. Quitting smoking improves CRC-specific survival (HR ≥ 10 years = 0.76; 95% CI = [0.67 to 0.85], HR is the hazard ratio) [36].

Obesity and a sedentary lifestyle are associated with the onset of colorectal cancer [37]. In our CRC group, more than half of the patients were obese and had A blood type followed by 0 blood type (Table 1). Similar results were previously published in the scientific literature, with CRC reported more frequently among subjects with A blood type [38], similar to gastric cancer [39]. 

The value of C-reactive protein was higher in the CRC group than in the control group (*p*-value < 0.0001), a result similar to what Holm et al. reported [40]. The patients with CRC had higher neopterin and chitotriosidase levels than the controls, but only neopterin reached the significance threshold (Figure 2). Elevated levels of circulating neopterin in patients with CRC compared to controls were previously reported [11,41]. The neopterin circulating levels in our study look higher (Table 2, Figure 2) than the values reported by Hacisevki et al. [41] (CRC group: 4.20 ± 0.68 ng/mL, *n* = 40 vs. control group: 1.57 ± 0.13 ng/mL, *n* = 25 in the controls). Circulating chitotriosidase shows similar values in CRC patients than controls (Table 2, Figure 2). Opposite to our results, Song et al. [42] reported higher chitinase values in patients with CRC than in controls in the Chinese population. The characteristics of the evaluated population could explain the differences. In Romania, at least one lipid abnormality is reported as 67.1%, and the prevalence of low HDL-cholesterol is 47.8% (95%CI = [46.3 to 49.2%]) [42]. High neopterin circulating levels have been reported as associated with low HDL-cholesterol levels (high-density lipoprotein) [43]. 

In our study, circulating values of chitotriosidase and neopterin proved similar in smokers and non-smokers regardless of the group (CRC or C group). As previously reported, smoking is not a confounder [21,22]. In neoplastic patients, the inflammation effect induced by exposure to nicotine could be masked by tumor-associated chronic inflammation. 

### 4.2. Changes of Evaluate Biomarkers at 1-Year Follow-Up in Patients with Colorectal Cancer

Chitotriosidase and neopterin circulating values decreased at 1-year follow-up (*p*-value < 0.002, Figure 3), showing the effect of tumor removal on these markers. The decreased levels of these biomarkers could also be explained by the systemic treatment (76.2% of the patients have undergone adjuvant chemotherapy and one patient, radiotherapy), which, combined with surgery, is expected to improve the survival rates. However, 19% of our patients with CRC exhibited tumor recurrence, and 23.8% had metastases (mainly liver, peritoneal, or bone). Li et al. [18] reported on the Chinese population based on SNP analysis that variants rs61745299 and rs35920428 in the CHIT1 gene that encode enzyme chitotriosidase are associated with the risk of CRC. 

The circulating values of CEA and CRP are associated with TNM stages, differentiation grade, presence of metastasis, and complications (Table 4). The changes in CEA and CRP circulating values 1 year after surgery (Table 4) suggest a decrease in tumor burden after surgery [44]. Similarly, circulating values of chitotriosidase and neopterin were significantly altered in association with tumor status at baseline and 1-year follow-up (Table 5, Figure 4 and Figure 5). Until now, studies have focused only on the difference between specific gene mutations and tumor status [45]. Thus, the results of our study shed light on possible, more cost-effective, and faster ways to predict the evolution of the tumor in patients with colorectal cancer.

Our results show that the postoperative trend of circulating chitotriosidase levels follows the carcinoembryonic antigen in a statistically significant monotonic moderate but statistically significant association. This result suggests the possible usefulness of this biomarker in evaluating disease progression, with increased levels compared to baseline in the presence of recurrence or metastasis. Moreover, this association implies that chitotriosidase could be used as a substitute biomarker in CEA non-secreting adenocarcinomas.

### 4.3. Study Limitations and Further Research

Several limitations of our study must be listed. The main limitations are related to the applied study design, which takes the causality analysis out of the discussion. In our study, the higher percentage of smokers in the CRC group compared to the control group is reflected in the inflammatory status of patients with CRC and the levels of evaluated biomarkers. So, the circulating levels of chitotriosidase and neopterin could not be solely attributed to colorectal cancer, similar to other tumor markers used in daily healthcare practice. However, the changes in the dynamic of the circulating levels of chitotriosidase and neopterin (pre- and post-surgery) support the possible use of these serum markers in the postoperative follow-up of patients with colorectal cancer. Furthermore, the number of evaluated patients is small, and the number of patients lost from observation is higher than expected and estimated. The overlap of our study with the COVID-19 pandemic, which limited access of patients with neoplasms to hospitals, could explain the high percentage of patients lost from follow-up. According to these limitations, the generalizability of the results is not possible. However, considering lipid profile and therapeutic schemas, our results support an extensive evaluation of chitotriosidase and neopterin circulating values as pre- and post-surgery markers. More comprehensive and controlled studies are needed to appropriately link the assessment of these biomarkers with patients’ and tumors’ characteristics. Furthermore, evaluating tissue chitotriosidase and neopterin levels could bring more insights into the effectiveness of these markers in assessing patients with colorectal cancer. 

## 5. Conclusions

Our study showed the value of circulating chitotriosidase and neopterin levels in distinguishing T-, N-, and M-stage before surgical treatment, with higher performances of chitotriosidase regarding differentiation grade. We observed an association between tumor recurrence or metastasis and high levels of circulating neopterin and chitotriosidase, with a counterbalance of significantly lower levels in patients with good evolution after surgery. The postoperative trend of circulating chitotriosidase levels follows the carcinoembryonic antigen in a statistically significant monotonic moderate but statistically significant association. This association supports further evaluation of chitotriosidase as a substitute biomarker in CEA non-secreting adenocarcinomas.

## Figures and Tables

**Table 1:** Demographic and clinical characteristics by groups.

	All (*n* = 88)	CRC Group (*n* = 44)	C Group (*n* = 44)	*p*-Value
Age, years ^a^	63 (10.6)	63.4 (11)	62.5 (10.3)	0.6982
Smoking, yes ^b^	32 (36.4)	22 (50)	10 (22.7)	0.0078
Alcohol, yes ^b^	22 (25)	13 (29.5)	9 (20.5)	0.3248
BMI, kg/m^2 c^	25 [23.8 to 28]	26 [22 to 29]	25 [24 to 26.3]	0.5259
BMI class ^a^				0.7549
Normal Overweight Obese	35 (39.8) 6 (6.8) 47 (53.4)	17 (38.6) 4 (9.1) 23 (52.3)	18 (40.9) 2 (4.5) 24 (54.5)	
Blood type ^b^				0.0123
0 A B AB	29 (33) 35 (39.8) 13 (14.8) 11 (12.5)	14 (31.8) 24 (54.5) 3 (6.8) 3 (6.8)	15 (34.1) 11 (25.0) 10 (22.7) 8 (18.2)	

^a^ results are expressed as mean (standard deviation), comparisons between groups by Student *t*-test; ^b^ results are expressed as no. (%); Chi-squared test or Fisher exact test (stat. = n.a., where n.a. = not available); ^c^ median [Q1 to Q3], where Q is the quartile; comparisons between groups by Mann-Whitney test.

**Table 2:** Values of circulating markers according to the groups.

Marker	CRC Group (*n* = 44)	C Group (*n* = 44)	*p*-Value
CRP, mg/dL	1.3 [0.48 to 4.43]	0.39 [0.2 to 0.7]	<0.0001
Chitotriosidase, ng/mL	2.9 [1.6 to 4.7]	2.3 [0.7 to 4.3]	0.1299
Neopterin, ng/mL	5.2 [2.3 to 8.3]	2.4 [2 to 5.3]	0.0150

Data are reported as median [Q1 to Q3], where Q is the quartile. CRC stands for colorectal cancer *p*-values are from the Mann-Whitney test.

**Table 3:** Distribution of stage, differentiation grade, and complications in patients with CRC at the inclusion in the study.

Characteristic	*n* (%)	Characteristic	*n* (%)
T stage		M stage	
T1/T2 T3 T4	10 (22.7) 29 (65.9) 5 (11.4)	M0 M1	35 (79.5) 9 (20.5)
N stage		G grade	
N0 N1 N2	15 (34.1) 18 (40.9) 11 (25)	G1 G2 G3	17 (38.6) 20 (45.5) 7 (15.9)
Complications, yes ^a^	31 (70.5)		

*n* = number of patients, % = percentage; ^a^ the most common complication was wound suppuration (6/31), followed by bleeding, prolonged ileus, acute urinary retention, or seroma, each occurring in 3/31 patients.

**Table 4:** Variation of CEA and CRP pre- (baseline) and postoperative (1-year follow-up) and between sub-groups.

Characteristic	CEA, ng/mL			CRP, mg/dL		
	Baseline	1-y Follow-Up	*p*-Value ^c^	Baseline	1-y Follow-Up	*p*-Value ^c^
T-stage						
T1T2, *n* = 10 T3, *n* = 29 T4, *n* = 3 *p*-value ^a^	5.4 [3.7 to 10.8] 5.2 [2.7 to 6.7] 8.4 [8.2 to 8.6] 0.0147 *	3.7 [2.2 to 4] 2.9 [1.7 to 5.6] 12 [11.7 to 35.7] 0.0553	0.6465 0.1059	1.4 [0.6 to 3.8] 1.2 [0.4 to 3.2] 2 [1.2 to 2.7] 0.5603	3.5 [0.9 to 5.7] 0.9 [0.4 to 2.2] 1.2 [1.1 to 2.1] 0.2122	0.4413 0.1494
N-stage						
N0, *n* = 15 N1, *n* = 17 N2, *n* = 10 *p*-value ^a^	5 [3.6 to 6.9] 5.6 [2.2 to 7.9] 5.7 [3.1 to 7.4] 0.7163	3.2 [2.1 to 4.1] 3.4 [1.8 to 5.8] 3.4 [1.8 to 11.6] 0.9890	0.2013 0.9588 0.9594	1.2 [0.4 to 4.7] 1 [0.5 to 2.5] 2.2 [0.7 to 12] 0.4440	1 [0.5 to 5.1] 0.9 [0.5 to 2.9] 0.8 [0.5 to 2.1] 0.6889	0.7299 0.8203 0.3329
M-stage						
M0, *n* = 35 M1, *n* = 7 *p*-value ^b^	5.6 [2.7 to 7.3] 5.2 [4 to 11.2] 0.0943	2.9 [1.8 to 4.1] 18 [9 to 25] 0.0098	0.0340 0.0630	1.2 [0.5 to 3.3] 1.2 [0.7 to 3.7] 0.5904	0.9 [0.4 to 3] 1.5 [0.8 to 4.1] 0.2877	0.2959 0.7532
Metastasis			n.a.			n.a.
Liver, *n* = 5 Others, *n* = 2	5 [3 to 8.4] 16.5 [10.8 to 22.1]	5.8 [4.2 to 12] 16.9 [11.3 to 22.4]		1.2 [1 to 1.4] 3 [2 to 3.9]	1.5 [1 to 2.9] 1 [0.8 to 1.3]	
G grade						
G1, *n* = 17 G2, *n* = 18 G3, *n* = 7 *p*-value ^a^	5.6 [2.7 to 7] 5.5 [2.7 to 7.4] 5.2 [3.4 to 9.9] 0.7331	2 [1.6 to 4.2] 3.7 [2.1 to 8.6] 5.8 [2.4 to 17.1] 0.2081	0.0929 0.9826 0.8658	1.2 [0.3 to 3.4] 2 [0.6 to 3.8] 1.2 [1.1 to 2.1] 0.6443	0.9 [0.3 to 1] 1.7 [0.5 to 3.2] 1.5 [0.9 to 2.3] 0.3367	0.3088 0.5701 0.6121
Complications						
Yes, *n* = 28 No, *n* = 13 *p*-value ^b^	5.6 [3.4 to 7.1] 3.8 [2.4 to 7.4] 0.3679	2.9 [1.8 to 5.6] 3.9 [2.2 to 9.6] 0.4793	0.1648 0.9165	2 [0.5 to 4.9] 1 [0.5 to 1.2] 0.1728	0.9 [0.4 to 3] 1.6 [1 to 3] 0.0970	0.0912 0.1730

CEA = carcinoembryonic antigen; CRP = C-reactive protein; ^a^ Kruskal-Wallis test; ^b^ Mann-Whitney test; * Mann-Whitney test T3 vs. T3: z-stat = −2.82, *p*-value = 0.0048; ^c^ Wilcoxon matched pairs test; 1-y = 1-year; n.a. = not applicable.

**Table 5:** Variation of chitotriosidase and neopterin according to different sub-groups.

Characteristics	Chitotriosidase (ng/mL)		Neopterin (ng/mL)	
	Baseline	Follow-Up	Baseline	Follow-Up
T-stage				
T1/T2, *n* = 10 T3, *n* = 29 T4, *n* = 3 *p*-value ^a^	2.1 [1 to 3.4] 2.6 [1.6 to 4.6] 4 [3.5 to 5.9] 0.0209 ^1a^	3.3 [1.6 to 4.3] 2 [1 to 4.1] 4.2 [2.7 to 4.3] 0.8331	2.8 [2 to 5.6] 5.1 [2.3 to 8.2] 8.8 [5.7 to 9.4] 0.0219 ^1b^	1.6 [1.3 to 2.1] 1.7 [1.4 to 2.1] 4 [3.1 to 6.1] 0.0609
N-stage				
N0, *n* = 15 N1, *n* = 17 N2, *n* = 10 *p*-value ^a^	1.3 [0.9 to 2] 3 [2.4 to 4] 5 [3 to 6] 0.0005 ^2a, 2d^	1.3 [0.6 to 3.3] 2.6 [1.2 to 4.2] 4.4 [3.1 to 4.7] 0.0249 ^2b, 2e, 2h^	2.1 [1.9 to 2.3] 5.6 [2.6 to 7.9] 8.5 [7.9 to 9.3] <0.0001 ^2f, 2i^	1.3 [1.2 to 1.4] 1.7 [1.6 to 2.3] 2.1 [1.9 to 3.2] 0.0001 ^2c,2g^
M-stage				
M0, *n* = 35 M1, *n* = 7 *p*-value ^b^	2.4 [1.3 to 3.4] 6.6 [6.3 to 7.3] <0.0001	1.5 [0.9 to 4] 5.3 [4.7 to 5.6] <0.0001	2.6 [2.2 to 6.7] 9.6 [9.2 to 9.6] <0.0001	1.6 [1.3 to 2] 4.9 [2.9 to 6.8] 0.0003
Metastasis				
Liver Others	6.6 [6.1 to 7.7] 6.7 [6.6 to 6.8]	4.7 [4.7 to 5.5] 5.5 [5.4 to 5.5]	9.6 [9.4 to 9.7] 9.3 [9.2 to 9.4]	5.5 [3.5 to 8.1] 3.5 [2.7 to 4.2]
G grade				
G1, *n* = 17 G2, *n* = 18 G3, *n* = 7 *p*-value ^a^	2.4 [1.2 to 3.2] 2.4 [1.4 to 4.3] 5 [4.3 to 6.3] 0.0136 ^3a^	1.4 [1 to 2.6] 3.3 [0.9 to 4.3] 4.7 [3.7 to 5] 0.0101 ^3b, 3c^	2.5 [2.1 to 7.8] 4.9 [2.4 to 7.2] 8.1 [5.1 to 8.9] 0.2628	1.5 [1.3 to 1.7] 1.9 [1.6 to 2.3] 2.3 [1.7 to 3.9] 0.0680
Complications				
Yes, *n* = 29 No, *n* = 13 *p*-value ^b^	2.5 [1.2 to 3.8] 3.4 [2.3 to 4.6] 0.3544	2 [1 to 4.3] 4 [1.4 to 4.7] 0.2313	5.1 [2.3 to 7.9] 4.8 [2.3 to 8.2] 0.8169	1.7 [1.4 to 2] 2.1 [1.3 to 2.9] 0.6243

Stat. = statistics of the test; ^a^ Kruskal-Wallis test; ^1a^ T1T2 vs. T4 (*p*-values = 0.0183); ^1b^ T1T2 vs. T4 (*p*-values = 0.0187); ^b^ Mann-Whitney test; ^2a^ N0 vs. N1: z-stat = −2.91, *p*-value = 0.0036; ^2b^ N0 vs. N1: −4.07 (*p*-value = 0.00005); ^2c^ N0 vs. N1: −3.81 (*p*-value = 0.0001); ^2d^ N0 vs. N2: −3.37 (*p*-value = 0.0007); ^2e^ N0 vs. N2: −2.47 (*p*-value = 0.0136); ^2f^ N0 vs. N2: −3.74 (*p*-value = 0.0002); ^2g^ N0 vs. N2: −3.36 (*p*-value = 0.0008); ^2h^ N1 vs. N2: −2.08 (*p*-value = 0.0372); ^2i^ N1 vs. N2: −2.13 (*p*-value = 0.0328); ^3a^ G1 vs. G3: −3.24, (*p*-value = 0.0012); ^3b^ G1 vs. G3: −3.18 (*p*-value = 0.0015); ^3c^ G2 vs. G3: −2.03 (*p*-value = 0.0426).

## Data Availability

The data presented in this study are available on request from the corresponding authors. The data are not publicly available due to privacy restrictions.
